# Pinhole Viewing Strengthens the Hollow-Face Illusion

**DOI:** 10.1177/0301006615599304

**Published:** 2015-08-31

**Authors:** Trent Koessler, Harold Hill

**Affiliations:** School of Psychology, University of Wollongong, NSW, Australia; School of Psychology, University of Wollongong, NSW, Australia

**Keywords:** hollow-face illusion, depth reversal, pinhole viewing, accommodation, focus blur, vergence

## Abstract

A hollow (concave) mask appears convex when viewed from beyond a certain distance even when viewed stereoscopically—this is the hollow-face illusion. At close viewing distances, the same mask is seen as hollow even when disparity information is eliminated by monocular viewing. A potential source of nonpictorial, monocular information that favors a veridical percept at close distances is accommodation in conjunction with focus blur. In this article, we used pinhole viewing to minimize this potential source of information and test whether it affects whether a hollow mask is seen as veridical (concave) or illusory (convex). Since monocular viewing also facilitates the illusory (convex) percept, it was included in the design both as a comparison and to test whether any effect of accommodation depends on vergence. Pinhole viewing was found favor the illusory percept, and its effect was at least as large as, and added to, that of monocular viewing. A control experiment using tinted glasses that attenuate illumination at least as much as the pinholes did not strengthen the illusion ruling out explanations in terms of reduced luminance. For pinhole viewing, there was no difference between monocular and binocular conditions. The results are interpreted as evidence that focus driven depth information affects perceived three-dimensional shape at close distances even when other sources of depth information are available. The lack of a difference between monocular and binocular pinhole viewing suggests that, by disrupting accommodation, pinholes may also interfere with linked vergence cues to depth.

## Introduction

A hollow face mask is seen as convex when viewed from beyond a certain distance, even when unambiguous stereoscopic depth information from binocular disparities is available. This is the hollow face illusion. When viewed from close enough, however, the same mask is seen as hollow even if viewing is monocular and binocular disparities are no longer available (Hill & Bruce, 1993; Papathomas & Bono, 2004). One source of nonpictorial depth information that might influence whether a hollow face is seen as veridical (concave) rather than illusory (convex) at close distances is focus blur driven accommodation. We tested this possibility using pinhole viewing which should minimize this potential source of information about real depth structure and thus increase the likelihood of seeing the hollow face as convex.

Accommodation potentially provides information about the absolute egocentric depth of the part of the visual scene that is being fixated, at least within the 2- to 3-m range over which it varies (Fisher & Ciuffreda, 1988; Howard, 2012). Accommodation and focus blur normally form a closed feedback loop that keeps the object of regard in focus. Blur itself is ambiguous with respect to depth because points in front and behind the region of focus can appear equally blurred. None the less the initial accommodative response to a step change in the depth of an accommodative stimulus is normally in the correct direction even in the absence of other depth cues (Campbell & Westheimer, 1959; Fincham, 1951). Correct direction appears to be specified by chromatic, spherical, and other aberrations or the Stiles-Crawford effect. The closed-loop feedback between changes in accommodation and changes in blur could also potentially provide information about depth. In practice, however, depth judgments based on accommodation do not appear to benefit from active switching in depth of fixation (Nguyen, Howard, & Allison, 2005). In general, the evidence that accommodation affects depth perception is relatively weak, leading Mon-Williams and Tresilian to conclude on the basis of their experimental evidence and review of the literature that “there appears to be little support for the notion that accommodation provides useful distance information” (Mon-Williams & Tresilian, 2000, p. 402).

In this experiment, we used pinhole glasses to open loop (Busby & Ciuffreda, 2005; Heath, 1956; Hennessy, Iida, Shina, & Leibowitz, 1976; Morgan, 1968; Ward & Charman, 1987), or at least quasiopen-loop (Frisby, Buckley, & Horsman, 1995), accommodation. Pinhole viewing is associated with an extended depth of field which reduces or eliminates blur and results in a tonic state of accommodation. With pinhole viewing, blur and accommodation no longer even potentially provide information about depth, either because they are eliminated from cue combination or because they now signal a flat surface (Watt, Akeley, Ernst, & Banks, 2005). In either case, accommodation would be neutral with respect to whether the hollow-face illusion appears convex or concave, the task required here. We used multiple pinhole glasses in order to minimize the reduction in retinal illuminance and to allow for changes in binocular fixation while still minimizing focus blur and accommodation as potential sources of depth information (Frisby et al., 1995).

Accommodation is synkinetically linked with vergence (Fincham, 1951; Fincham & Walton, 1957; Morgan, 1968; Semmlow & Heerema, 1979) which is itself another potential ocular cue to depth. The evidence that vergence contributes to human depth perception is stronger than that for accommodation (Tresilian, Mon-Williams, & Kelly, 1999). In depth reversal illusions, the steady state vergence is typically to a point in front of actual surface, consistent with observers verging to perceived depth (Hoffmann & Sebald, 2007; Wagner, Ehrenstein, & Papathomas, 2009). This requires that feedback from binocular disparity, the usual stimulus for vergence, is overridden. We included both monocular and binocular viewing conditions so as to be able to test the effect of accommodation both with and without disparity driven vergence.

We used “flipping distance,” the distance at which perception of the hollow mask changes from convex to concave, as a measure of the strength of the illusion (Hill & Bruce, 1993). Viewing distance affects the magnitude of most if not all sources of information about depth, with the greatest changes occurring within the first few meters of the observer. This is also the range within which accommodation and vergence are required and within which the mask is seen as hollow. While this codependence of multiple depth cues on distance complicates interpretation of flipping distance, differences between viewing conditions can be used to infer whether or not a manipulated source of depth information helps disambiguate the illusion. Work with reverspectives shows that viewing distance, rather than the absolute disparity magnitudes, is critical in determining reversibility (Dobias & Papathomas, 2013).

In summary, we used pinhole viewing to reduce or eliminate focus blur driven accommodation as a potential source of disambiguating depth information. If this source of depth information normally plays a role in disambiguating the hollow-face illusion at close distances, we would expect shorter flipping distances when pinhole viewing. We included monocular and binocular viewing for comparison and to test whether any effect of accommodation depends on vergence.

## Method

### Participants

Twenty undergraduate psychology students (12 females, 8 males) between the ages of 18 and 40 (mean 21.5) took part in the experiment for course credit. The experiment was approved by the University of Wollongong Human Research Ethics Committee (ref HE12/462).

### Materials

The mask used was the unpainted 35.5 cm × 45.5 cm white plastic “Shakespeare Hollow Face” available from http://www.grand-illusions.com. Commercially available multiple pinhole glasses were used which consisted of perforated opaque nylon “lenses” each with 114 pinholes aligned in an offset grid of nine rows (http://www.shopsodial.com/SODIAL-TM-Eyesight-Improvement-Exercise/dp/B008SK5CWK). Pinholes were 1.1 mm in diameter and separated by 2.5 mm. The “lenses” were 1.1 mm thick. The pinhole arrays were fitted in black plastic spectacle frames 14.5 cm wide, 4.2 cm high. The hollow face was lit from above and behind with a goose-necked Krompton halogen lamp (KL2379) which uses two bi-pin 50 W natural color bulbs in an otherwise black and dark room. The luminance of the mask as measured using a spot photometer pointed at the bridge of the nose from a distance of 1 m was 157 cd/m^2^. The mask was placed 1.3 m above the ground, that is, at approximately head height facing the participant’s direction of movement. Flipping distance was measured from the base of the mask.

### Procedure

Spatial acuity was measured using a standard seven-letter Snellen chart (http://www.eyerobics.com.au/eye_chart.html) and stereo acuity using the “stereo-butterfly” stereoacuity kit (http://www.stereooptical.com/products/stereotests). All acuity testing was undertaken both with and without pinholes. Spatial acuity was measured both binocularly and monocularly. Visual acuity was tested at 1.8 m and scored as Snellen decimals. A Snellen decimal of 1.0 corresponds to 6/6 vision with higher values indicating better acuity. The proportion correct of the next most difficult line reported was linearly interpolated to give intermediate scores.

After acuity testing, participants were familiarized with the mask to determine if they experienced the illusion. All did. Participants were also asked to experiment with viewing the mask from different distances and to choose a criterion for deciding when appearance changed between convex and concave. They were encouraged to check their percept by observing whether the face or mask followed them when they moved from side-to-side consistent with the perception of an illusory convex face, or remained stationary consistent with the perception of a hollow (concave) face.

For experimental trials, participants either advanced toward the mask starting approximately 3 m away or slowly retreated from 0 m until their perception of mask depth changed from convex to concave or vice versa, respectively. More details on this method can be found in Hill & Bruce (1993). There were 16 trials in the main experiment presented in a different random order for each observer. Two control trials followed the experiment proper, with illumination halved by turning one of the bulbs off. These control trials were conducted binocularly without pinholes.

### Design

A 2 Pinholes (with/without) × 2 Eyes (monocular/binocular) × 2 Direction (approaching/retreating) fully factorial within subjects design was used. There were two repetitions for each cell. The dependent variable was flipping distance in millimetres.

## Results

### Flipping Distance

The distribution of data points did not differ significantly from normal in any of the conditions, and variance did not violate the assumption of homogeneity. There was no effect of repetition, and data were collapsed across this factor.

**Figure 1.**
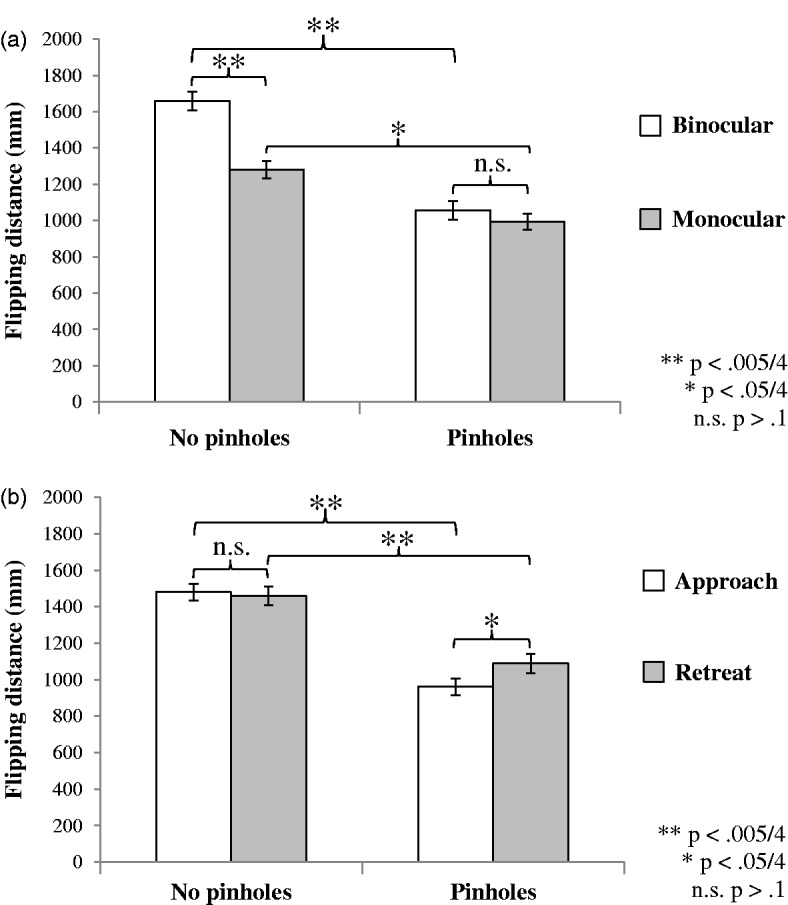
(a): The statistically significant Pinholes × Binocularity interaction. (b): The statistically significant Pinholes × Direction of movement interaction. All error bars show standard error of mean after within subject variance is removed.

A 2 Pinholes (with/without) × 2 Eyes (monocular/binocular) × 2 Direction (approaching/retreating) fully factorial repeated measures analysis of variance revealed significant interaction effects between number of eyes and pinhole viewing, *F*(1, 19) = 17.02, *p* < .001, partial-η^2 ^= .47, and between pinhole viewing and direction of movement, *F*(1, 19) = 5.54, *p* = .03, partial-η^2 ^= .23. These interactions are plotted in Figure 1(a) and (b) respectively. The interactions modulated significant main effects of eyes, *F*(1, 19) = 18.29, *p* < .001, partial-η^2 ^= .49, and pinholes, *F*(1, 19) = 37.92, *p* < .001, partial-η^2 ^= .67. Both monocular and pinhole viewing significantly reduced flipping distance overall. No other main effects or interactions were significant (all *p*’s > .1).

Follow-up analysis of the pinholes × eyes interaction showed pinhole viewing significantly reduced flipping distance for both monocular and binocular viewing whereas monocular viewing only significantly reduced flipping distance for nonpinhole viewing. For the pinholes × direction interaction, the effect of direction was only significant for pinhole viewing. The effect of pinholes was significant for both directions of movement. These significant differences are indicated in Figure 1 (a) and (b).

### Luminance controls

1. The half luminance control trials were not significantly different from the equivalent binocular nonpinhole conditions conducted as part of the main experiment (*p* > .1).
2. A follow-up experiment was conducted using tinted glasses chosen to match the reduction in light reaching the eye associated with pinholes (cf. Frisby et al., 1995). The luminance of the mask measured using a spot photometer pointed at the bridge of the nose from a distance of 1 m was 25 cd/m^2^ through the tinted glasses and 26 cd/m^2^ through the pinholes (compared with 157 cd/m^2^ unrestricted). Run as a 3 Glasses (tinted/pinhole/none) × 2 Eyes (monocular/binocular) × 2 Direction (approaching/retreating) fully factorial repeated measures design with 12 participants (eight female), analysis of variance gave an independent main effect of type of glasses, *F*(2, 22) = 22.68, *p* < .001, partial-η^2 ^= .67, and of number of eyes, *F*(2, 22) = 9.41, *p* = .011, partial-η^2 ^= .46. Follow-up pairwise comparisons using Bonferroni correction on the effect of glasses showed that flipping distance (mm) was significantly less for pinholes (*M* = 1068, *SE* = 12) than for either tinted glasses (*M* = 1359, *SE* = 15, *p* = .006) or normal viewing (*M* = 1523, *SE* = 13, *p* < .001). Tinted and normal did not differ significantly from each other (*p* = .199). The effect of eyes was as expected with flipping distance lower for monocular (*M* = 1165, *SE* = 14) than for binocular (*M* = 1468, *SE* = 13) viewing. No other main effects or interactions were significant (all *p*’s > .1). In particular, the interactions of glasses with number of eyes *F*(2, 22) = 2.08, *p* = .148, partial-η^2 ^= .16, and direction *F*(2, 22) = 2.10, *p* = .147, partial-*η^2^*^ ^= .16, were not significant in this control experiment (even when the tinted glasses condition was excluded from the analysis). This may have been in part due to the lower number of participants, 12 as opposed to 20. Consistent with the pattern reported in the main experiment of pinholes disrupting stereo, the difference between monocular and binocular viewing was less for pinholes (*M* = 13, *SE* = 17) than for tinted glasses (*M* = 31, *SE* = 12) or normal (*M* = 46, *SE* = 12). Pairwise comparisons to check consistency with the main experiment showed an effect of number of eyes for normal, *t*(11) = 3.93, *p = *.002, and tinted *t*(11) = 2.71, *p = *.020, but not for pinholes, *t*(11) = 0.76, *p = *.463. Also in line with the main experiment, the difference between approaching a retreating directions was greater for pinhole viewing (*M* = −35, *SE* = 11, i.e., approaching less) that for normal (*M* = −16, *SE* = 17) or tinted glasses (*M* = −13, *SE* = 15). Pairwise comparisons were again consistent with the main experiment showing a significant difference between approaching and retreating for pinholes, *t*(11) = 3.15, *p = *.009, but not normal, *t*(11) = 0.97, *p = *.355, or for tinted glasses, *t*(11) = 0.84, *p = *.417.

### Acuity tests

Neither spatial nor stereo acuity data were normal, and both were analysed nonparametrically. One participant was off the scale for both stereo and visual acuity for binocular pinhole viewing and was excluded from these analyses. Including or excluding their flipping distance data from analysis of the main experiment reported above made no difference to the pattern of results.

A nonparametric Wilcoxon signed rank test showed that stereoacuity was slightly but significantly better without pinholes (median = 40 arcs), than with (median = 50 arcs), *Z* = 2.51, *p* = .012.

For visual acuity, a 2 Pinholes × 2 Eyes resampling analysis of the nonnormal data with 5,000 repetitions showed a main effect of Pinholes, *F*(1, 18) = 19.28, *p* < .001: acuity with pinholes was *M* = 1.18, *SE* = 0.02; without pinholes was *M* = 1.30, *SE* = 0.02. There was also a main effect of number of eyes *F*(1, 18) = 5.76, *p* = .032: monocular acuity was *M* = 1.22, *SE* = .02; binocular acuity was *M* = 1.27, *SE* = 0.02. The main effects of both pinhole and monocular viewing correspond to a reduction in acuity. There was no interaction between pinholes and number of eyes, *p* = .700.

## Discussion

Both monocular and pinhole viewing strengthened the hollow-face illusion. The effect of pinholes is consistent with focus blur driven accommodation helping to disambiguate perceived three-dimensional (3-D) shape in a situation where other sources of depth information are available. The effect of monocular viewing was as expected and confirms that binocular disparities help disambiguate the illusion at close distances. There was an effect of pinhole viewing on monocular as well as binocular viewing—the flipping distance was reduced indicating a stronger illusion. This suggests that pinhole viewing reduces the depth information about the actual concave surface of the mask under both monocular and binocular viewing.

The claim that accommodation affects depth perception contradicts the widely accepted conclusion that the human visual system makes little or no use of this potential source of depth information. The task used here was very different from the explicit judgements of absolute egocentric distance under reduced cue conditions that provide the evidence that accommodation in itself is not a strong cue to depth (Fisher & Ciuffreda, 1988; Mon-Williams & Tresilian, 2000). In the present experiment, ordinal depth would be sufficient for judging whether the face “sticks out” or not. The judgment is only about the relative depth of the face (concave or convex) and logically independent of distance from the observer. This raises the possibility that the depth cues affected by pinhole viewing could influence 3-D shape perception even if they do not influence perceived distance with respect to the observer.

This experiment also differs from much previous work in that we did not attempt to present accommodative depth information is isolation but rather to remove it from a situation where multiple other sources of depth information remained available. This approach, and the indirect measure of perceived depth, is more similar to Holway and Boring’s (1941) experiment where pinhole viewing was also used to disrupt depth perception and found to affect perceived size constancy. In both cases, the findings are contrary to the argument that accommodation would not influence even ordinal depth under full-cue conditions if it is unreliable and thus given a low weight in depth cue combination (Mon-Williams & Tresilian, 2000). Instead accommodation may have a critical role in combination with other sources of depth information, for example, helping to disambiguate them, even if it is not effective in isolation.

Alternative explanations for the effect of pinholes include the associated reduction in retinal illuminance. A control involving halving the illumination of the mask had no effect on flipping distance but the reduction in illuminance due to pinholes is by more than a factor of two. However, a second control experiment using tinted glasses that reduced the light reaching the eye by slightly more than the pinholes had no effect on flipping distance, ruling out this explanation. Frisby et al. (1995) also used dark sun glasses as a control for their estimated 94% attenuation of incident light by pinholes and similarly concluded that “the effect of the pinhole arrays was not due to light attenuation” (p. 191). Multiple pinhole glasses can also result in two or more images of each point when pupils are dilated by more than the separation between pinholes (Colicchia, Hopf, Wiesner, & Zollman, 2008) which could also have disrupted depth processing. Again this possibility cannot be entirely ruled out but no participant complained of double vision during the experiment, and the effect is normally eliminated by a small change in view. Frisby et al. (1995) report equivalent results for single and multiple pinholes.

The effect of monocular viewing on depth reversibility was as expected and is as has been reported before (Hill & Bruce, 1993; Papathomas & Bono, 2004; Rogers & Gyani, 2010). Monocular viewing eliminates binocular retinal disparities as a potential source of depth information, and this makes it more likely that observers perceive the illusory, hollow face at closer viewing distances. Novel and unexpected was the lack of a difference between monocular and binocular pinhole viewing. While this may reflect a “floor” effect in the sense of a lower limit of flipping distance, that would require that pinhole but not monocular viewing reduces performance to floor. There was also small but significant reduction in stereo acuity associated with pinhole viewing. However, the stereo acuity testing also clearly showed that pinholes clearly did not entirely eliminate the use of binocular disparities in the way that that monocular viewing does. Instead, pinhole viewing may disrupt binocular depth perception by “interfering with the accommodation/vergence synkinesis” (Frisby et al., 1995, p. 192) rather than through “diminished stereo acuity” (Frisby et al., 1995, p. 189). Blur may directly drive vergence away from fixation due to limitations in processing large disparities (Held, Cooper, & Banks, 2012), but this signal to correct vergence would be absent during pinhole viewing. Vergence may also be accorded less weight in cue combination when accommodation is open loop and no longer available to provide an estimate of fixation distance (Watt et al., 2005). If pinhole viewing reduces binocular flipping distance because it interferes with vergence, this would also suggest that it may be vergence rather than binocular stereopsis that helps disambiguate the illusion viewed monocularly. In the second luminance control experiment, the interaction between monocular or binocular viewing and pinhole or normal viewing was not significant although there again appeared to be less if any difference between monocular and binocular for pinholes. This relationship between pinhole viewing and stereopsis appears worthy of further investigation.

The effect of pinhole viewing also interacted with direction of movement. Previously experiments have shown that the flipping distance for approaching observers is lower (i.e., closer) than that for retreating observers which is consistent with a hysteresis effect whereby the starting percept is maintained (Hill & Bruce, 1993; Rogers & Gyani, 2010). This effect was replicated here for pinhole, but not nonpinhole, viewing. This effect was not of primary concern here and is reported mainly for completeness.

The accuracy of accommodation depends on both the spatial frequency and the contrast of the stimulus (Fisher & Ciuffreda, 1988). The hollow-face illusion is relatively low contrast and lacks high frequency features such as sharp contours and so probably constitutes a relatively poor accommodative stimulus. If accommodation is important in resolving depth, this would in part explain the effectiveness of the illusion (Hill, Palmisano, & Matthews, 2012). An unanswered question is what depth information disambiguates the 3-D hollow face viewed monocularly through pinholes at short viewing distances? Motion parallax and perspective are two possibilities.

In summary, the large effect of pinholes on flipping distance suggests that they disrupt a source or sources of depth information that are usually critical for disambiguating the mask at close distances. An effect of pinholes on focus blur, accommodation and, indirectly, vergence provide a possible account for the pattern of results observed. This would suggest that focus blur and accommodation do play a role in 3-D shape perception under full cue conditions.
